# Comparison Between Closed-Loop Insulin Delivery System (the Artificial Pancreas) and Sensor-Augmented Pump Therapy: A Randomized-Controlled Crossover Trial

**DOI:** 10.1089/dia.2020.0365

**Published:** 2021-02-25

**Authors:** Ahmad Haidar, Laurent Legault, Marie Raffray, Nikita Gouchie-Provencher, Peter G. Jacobs, Anas El-Fathi, Joanna Rutkowski, Virginie Messier, Rémi Rabasa-Lhoret

**Affiliations:** ^1^ Department of Biomedical Engineering, McGill University, Montreal, Canada.; ^2^ Centre for Translational Biology, Research Institute of McGill University Health Centre, Montréal, Canada.; ^3^ Department of Pediatrics, Division of Endocrinology and Metabolism, McGill University Health Centre, Montréal, Canada.; ^4^ Centre for Outcomes Research and Evaluation, Research Institute of McGill University Health Centre, Montréal, Canada.; ^5^ Metabolic Diseases Research Unit, Institut de recherches cliniques de Montréal, Montréal, Canada.; ^6^ Department of Biomedical Engineering, Oregon Health and Science University, Portland, Oregon, USA.; ^7^ Nutrition Department, Faculty of Medicine, Université de Montréal, Montréal, Canada.; ^8^ Montreal Diabetes Research Center and Endocrinology Division, Montréal, Canada.

**Keywords:** Closed-loop, Artificial pancreas, Randomized-controlled trial, Sensor-augmented pump therapy

## Abstract

***Objective:*** Several studies have shown that closed-loop automated insulin delivery (the artificial pancreas) improves glucose control compared with sensor-augmented pump therapy. We aimed to confirm these findings using our automated insulin delivery system based on the iPancreas platform.

***Research Design and Methods:*** We conducted a two-center, randomized crossover trial comparing automated insulin delivery with sensor-augmented pump therapy in 36 adults with type 1 diabetes. Each intervention lasted 12 days in outpatient free-living conditions with no remote monitoring. The automated insulin delivery system used a model predictive control algorithm that was a less aggressive version of our earlier dosing algorithm to emphasize safety. The primary outcome was time in the range 3.9–10.0 mmol/L.

***Results:*** The automated insulin delivery system was operational 90.2% of the time. Compared with the sensor-augmented pump therapy, automated insulin delivery increased time in range (3.9–10.0 mmol/L) from 61% (interquartile range 53–74) to 69% (60–73; *P* = 0.006) and increased time in tight target range (3.9–7.8 mmol/L) from 37% (30–49) to 45% (35–51; *P* = 0.011). Automated insulin delivery also reduced time spent below 3.9 and 3.3 mmol/L from 3.5% (0.8–5.4) to 1.6% (1.1–2.7; *P* = 0.0021) and from 0.9% (0.2–2.1) to 0.5% (0.2–1.1; *P* = 0.0122), respectively. Time spent below 2.8 mmol/L was 0.2% (0.0–0.6) with sensor-augmented pump therapy and 0.1% (0.0–0.4; *P* = 0.155) with automated insulin delivery.

***Conclusions:*** Our study confirms findings that automated insulin delivery improves glucose control compared with sensor-augmented pump therapy.

ClinicalTrials.gov no. NCT02846831.

## Introduction

Type 1 diabetes is a chronic disease caused by the interaction of genetic determinants and environmental factors resulting in an autoimmune destruction of pancreatic beta cells. Intensive insulin therapy aiming at good glycemic control significantly reduces microvascular and macrovascular complications.^1,2^ However, hypoglycemia remains the major barrier to achieve glycemic targets,^3^ and more than 70% of people with type 1 diabetes do not achieve glycemic targets despite advances in insulin analogues, educational programs, insulin pumps, and glucose sensors.^4^

Automated insulin delivery systems are recent technologies that automate insulin pump delivery based on glucose sensor readings and a dosing algorithm.^5^ Several groups have developed dosing algorithms for automated insulin delivery systems,^6^ three of which were compared with sensor-augmented pump therapy in randomized day-and-night trials in outpatient unsupervised settings with no remote monitoring. The Cambridge algorithm was tested in several trials,^7–9^ including in participants with suboptimal^8^ and good glycemic control,^9^ and consistently showed improvements in glucose control. The University of Virginia's algorithm was also tested in several clinical trials,^10–12^ and showed improvements in glucose control, although the benefits were lower when the algorithm was embedded in a phone as opposed to a pump.^11,12^ The Medtronic first-generation dosing algorithm was tested in several nonrandomized trials,^13^ but their second-generation algorithm was recently tested in a 4-week randomized trial,^14^ and also showed improvements in glucose control.

Here, we present results of a randomized trial, comparing our automated insulin delivery system with sensor-augmented pump therapy over 12 days in outpatient, unsupervised, and free-living conditions with no remote monitoring. The system used the iPancreas platform used in other studies,^15–17^ and a model predictive control algorithm that was less aggressive than our earlier dosing algorithm,^18–20^ to prioritize patients' safety over optimal system performance in our first outpatient study.

## Methods

### Study design

We conducted an open-label, two-center, randomized crossover study in type 1 diabetes to compare an automated insulin delivery system with sensor-augmented pump therapy. Each intervention lasted 12 days in outpatient free-living settings with no remote monitoring. The washout period between the two interventions was a median of 17 (interquartile range [IQR] 16–22) days.

### Participants

From January 2019 to January 2020, participants were enrolled at the Montreal Clinical Research Institute and the Research Institute of McGill University Health Centre, Montreal, Canada. Participants were required to be older than 18 years, diagnosed with type 1 diabetes for at least 1 year, and using an insulin pump for at least 3 months. Exclusion criteria included clinically significant nephropathy, neuropathy, or retinopathy, acute macrovascular event within 6 months of screening, chronic use of acetaminophen, pregnancy, severe hypoglycemia within 2 weeks of screening, and diabetes ketoacidosis within 3 months of screening. Participants were required to remain within a travel distance of 2 h from Montreal during the interventions. Participants provided written informed consent. The study was approved by the local institutions' ethics committees.

### Randomization and masking

We used blocked randomization to generate allocation sequences, which were disclosed after the admission visit. Participants and investigators were not blinded to the allocation.

### Study procedures

After the study admission visit, participants had a run-in period of 12 days with a glucose sensor (Dexcom G5^®^; Dexcom, CA) and using their own pump. After 6 and 12 days of the run-in period, a member of the team reviewed participants' pump and sensor data, and adjusted their carbohydrate ratios or basal rates if significant hyperglycemia or hypoglycemia was observed.

On the sensor-augmented pump therapy arm, participants used Dexcom glucose sensor and their own pump for 12 days. On the automated insulin delivery arm, participants used the iPancreas system (Oregon Health and Science University, OR) for 12 days. The system consists of a Dexcom glucose sensor, a noncommercial t:slim TAP3 insulin pump (Tandem Diabetes Care, CA), and a cell phone (Nexus 5; LG Electronics). On the first day of the automated insulin delivery intervention, participants were admitted to our clinical research facilities in the morning, during which they were trained on the system usage, and were discharged in the afternoon. Participants were asked to switch to sensor-augmented pump therapy when driving on the first day of the intervention.

The automated insulin delivery system was initialized using participants' total daily insulin dose, carbohydrate ratios, and programmed basal rates. A new basal rate was calculated every 10 min based on a model predictive control dosing algorithm,^18–20^ which used the sensor data as an input. The computed basal rate was wirelessly communicated to the pump. The system had one adjustable glucose target between 6.0 and 7.0 mmol/L for the basal rate changes. Participants determined the target value and could change it anytime during the study. Participants were made aware of the exercise feature in the system, which would raise the glucose target by 3 mmol/L. As this was the first unsupervised outpatient study with this algorithm, internal parameters of the model predictive control dosing algorithm were tuned to make the algorithm less aggressive than earlier versions.

Participants were instructed to manually enter the carbohydrate content of the meals and snacks into the system, which calculated the prandial boluses. Participants could also deliver manual correction boluses through the system at anytime. The system had one adjustable glucose target between 6.0 and 7.0 mmol/L for the correction boluses, which were set and adjusted by participants. The system does not administer automatic boluses outside mealtimes. The system will switch to open-loop mode (delivering participant's usual basal rates) if communication with pump and sensor is lost for more than 20 and 30 min, respectively.

For both interventions, sensor alarm thresholds were determined by participants. Participants were contacted on the first evening of both interventions, as well as on days 3 and 9 to discuss any unexpected events or technical problems. Study teams were on call throughout the interventions to provide technical support. Participants were asked to treat hypoglycemia as per their standard practice.

### Study outcomes

The primary outcome was the percent of time that the sensor glucose readings were in the target range (3.9–10.0 mmol/L).^21^ Secondary outcomes included times spent below and above target range, glucose at 06:00, and glucose variability.^21^ Outcomes were calculated for the entire 12-day study period. For the automated insulin delivery intervention, outcomes included all available data, including when the system was in open-loop mode.

### Statistical analysis

We anticipated that automated insulin delivery would increase the percentage of time-in-target range by an absolute 15% (standard deviation [SD] = 22%) compared with sensor-augmented pump therapy. Therefore, using the formula for the paired *t*-test with 5% significance level, we calculated that 36 participants would provide a power of 80% for the trial.

A linear mixed model was fitted to the data while adjusting for the period effect. To examine for carryover effect, a model was fitted with the treatment by period interaction term. Residual values were examined for normality, and if skewed, the data were transformed using the square root function. *P*-values lower than 0.05 were regarded as significant. Results are reported as median (IQR) or mean (SD).

## Results

**FIG. 1.**
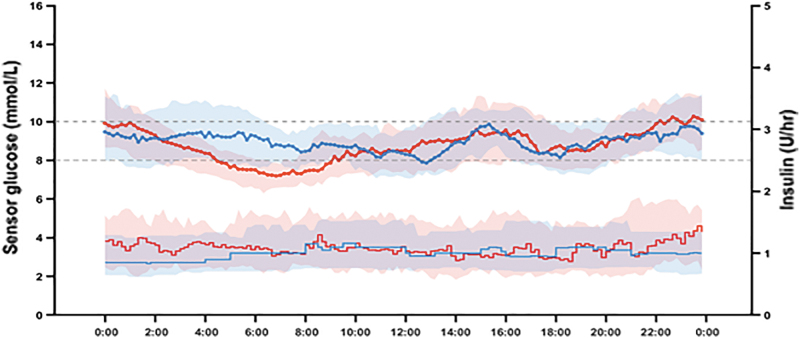
The median (IQR) profiles of individual mean glucose levels (*top*) and individual mean basal insulin deliveries (*bottom*) during 12-day automated insulin delivery (*red*; *n* = 36) and 12-day sensor-augmented pump therapy (*blue*; *n* = 36) interventions. At each time point, mean values were calculated for each participant, and then, the median (IQR) was calculated across participants. IQR, interquartile range.

Thirty-six participants were enrolled in the study and completed the two interventions (61% female, mean age 40 (16) years, HbA1c 7.5% (0.9%) [58 (10) mmol/mol], duration of diabetes 22 (13) years, total daily insulin 49 (22) U, 0.65 (0.22) U/kg; Table 1). Mean basal rate at the end of the run-in period was 1.1 U/h. The mean carbohydrate-to-insulin ratio at the end of the run-in period was 9.1 g/U. Glucose sensor readings were available 91.6% of the time during the automated insulin delivery interventions and 92.5% of the time during the sensor-augmented pump therapy intervention. During the automated insulin delivery intervention, the system was operational in closed-loop mode 90.2% of the time. Figure 1 compares the sensor glucose profiles of the two interventions.

**Table 1.** Baseline Characteristics of Study Participants (*n* = 36)

	Mean (SD)	Range (min.–max.)
Age, years	39 (16)	18–71
Duration of diabetes, years	23 (13)	7–53
Female participants, *n* (%)	22 (61)	–
Total daily insulin dose, U/day [U/kg/day]	49.5 (21.3) [0.65 (0.22)]	14.0–108.9 [0.19–1.16]
BMI, kg/m^2^	26.5 (4.9)	18.9–41.7
HbA1c, % [mmol/mol]	7.5 (0.9) [58 (10)]	5.2–9.8 (34–84)

BMI, body mass index; SD, standard deviation.

The automated insulin delivery system increased the median percentage of time spent in target range compared with sensor-augmented pump therapy from 61% to 69% (*P* = 0.006) and reduced median time spent below 3.9 mmol/L from 3.5% to 1.6% (*P* = 0.0021). Median time spent above 10.0 mmol/L was 36% with sensor-augmented pump therapy and 29% with automated insulin delivery (*P* = 0.052; Table 2) and glucose level at 06:00 was 9.3 and 7.5 mmol/L (*P* < 0.001; Table 2), respectively. Differences in glucose coefficient of variance, SD, and mean glucose level were not statistically significant (Table 2). No difference was observed in the primary outcome due to the order of interventions (data not shown). No difference was observed between the outcomes of week 1 and week 2 in either arm (data not shown). There was no severe hypoglycemia or ketoacidosis in either intervention.

**Table 2.** Comparisons Between the Automated Insulin Delivery and Sensor-Augmented Pump Therapy

	Sensor-augmented pump therapy (*n* = 36)	Automated insulin delivery (*n* = 36)	Paired difference,^a^*P*
Time spent at glucose levels (%)			
3.9–10.0 mmol/L	61 (53 to 74)	69 (60 to 73)	7 (1 to 10), 0.0058
3.9–7.8 mmol/L	37 (30 to 49)	45 (35 to 51)	7 (−2 to 13), 0.011
<3.9 mmol/L	3.5 (0.8 to 5.4)	1.6 (1.1 to 2.7)	−1.3 (−2.6 to 0.6), 0.0021
<3.3 mmol/L	0.9 (0.2 to 2.1)	0.5 (0.2 to 1.1)	−0.3 (−1.3 to 0.2), 0.0122
<2.8 mmol/L	0.2 (0.0 to 0.6)	0.1 (0.0 to 0.4)	0 (−0.4 to 0.1), 0.155
>7.8 mmol/L	58 (48 to 70)	52 (48 to 64)	−6 (−11 to 3), 0.061
>10.0 mmol/L	36 (23 to 43)	29 (24 to 38)	−6 (−9 to 2.6), 0.053
>13.9 mmol/L	10 (4 to 17)	7 (5 to 10)	−3 (−4 to 1), 0.068
Mean glucose (mmol/L)	9.2 (8.2 to 9.7)	8.8 (8.3 to 9.3)	−0.4 (−0.8 to 0.3), 0.18
Glucose at 06:00 (mmol/L)	9.3 (7.7 to 10.3)	7.5 (6.5 to 8.0)	−1.6 (−2.5 to −0.2), <0.001
SD of glucose (mmol/L)	3.3 (3.0 to 3.8)	3.2 (2.9 to 3.6)	−0.1 (−0.5 to 0.3), 0.13
CV of glucose (%)	37.2 (33.5 to 41.0)	36.1 (32.6 to 40.5)	−0.6 (−3.9 to 3.5), 0.48
Basal insulin (U/day)	23.3 (18.5 to 33.1)	26.3 (18.2 to 39.5)	3.1 (0.2 to 4.7), <0.001
Bolus insulin (U/day)	23.5 (14.9 to 33.0)	21.4 (16.6 to 31.3)	−2.2 (−5.0 to 1.6), 0.02

Data are median (IQR).

^a^ Automated insulin delivery minus sensor-augmented pump therapy.

CV, Coefficient of variation; IQR, interquartile range.

The benefits of automated insulin delivery in increasing the time-in-target range were primarily due to improvement in glucose control during the night. During the night (00:00–06:00) and during the day (06:00–00:00), automated insulin delivery increased median time-in-target range from 60% to 69% (*P* = 0.00074) and from 64% to 67% (*P* = 0.04), respectively (Table 3). The benefits of automated insulin delivery in decreasing the time below 3.9 mmol/L were primarily due to improvements during the day. During the day, automated insulin delivery decreased time below 3.9 mmol/L from 2.6% to 1.8% (*P* = 0.0019, Table 2). During the night, the time below 3.9 mmol/L was low in both interventions (1.4%–1.5%, Table 3). Mean glucose level was reduced during the night but not during the day (Table 3).

**Table 3.** Comparisons Between the Automated Insulin Delivery and Sensor-Augmented Pump Therapy During the Day (06:00–00:00) and Night (00:00–06:00)

	Sensor-augmented pump therapy (*n* = 36)	Automated insulin delivery (*n* = 36)	Paired difference,^a^*P*
Day outcome (06:00–00:00)			
Time spent at glucose levels (%)			
3.9–10.0 mmol/L	64 (52 to 76)	67 (60 to 76)	5 (−2 to 10), 0.041
3.9–7.8 mmol/L	39 (32 to 49)	42 (36 to 51)	5 (−4 to 12), 0.090
<3.9 mmol/L	2.6 (1.0 to 6.2)	1.8 (1.1 to 2.8)	−1.5 (−3.1 to 0.3), 0.0019
<3.3 mmol/L	0.8 (0.3 to 2.2)	0.4 (0.2 to 1.1)	−0.4 (−1.4 to 0.3), 0.010
<2.8 mmol/L	0.2 (0.0 to 0.6)	0.1 (0.0 to 0.5)	−0.1 (−0.4 to 0.1), 0.13
>7.8 mmol/L	58 (43 to 66)	56 (46 to 63)	−4 (−10 to 5), 0.29
>10.0 mmol/L	33 (20 to 45)	31 (21 to 37)	−4 (−8 to 3), 0.23
>13.9 mmol/L	9 (4 to 18)	8 (4 to 12)	−2 (−5 to 1), 0.23
Mean glucose level (mmol/L)	9.0 (8.0 to 9.9)	8.8 (8.1 to 9.4)	−0.2 (−0.7 to 0.4), 0.57
SD of glucose (mmol/L)	3.3 (2.8 to 3.9)	3.3 (2.7 to 3.6)	0.0 (−0.4 to 0.3), 0.33
CV of glucose (%)	36.6 (34.2 to 41.9)	36.3 (32.7 to 39.2)	0.0 (−5.0 to 2.5), 0.35
Basal insulin (U/18-h daytime)	17.7 (14.2 to 26.0)	19.4 (13.6 to 30.2)	−1.5 (−0.2 to 2.7), 0.0012
Bolus insulin (U/18-h daytime)	22.6 (14.1 to 30.8)	21.3 (16.5 to 29.7)	−1.8 (−3.1 to 1.2), 0.064
Night outcome (00:00–06:00)			
Time spent at glucose levels (%)			
3.9–10.0 mmol/L	60 (43 to 75)	69 (59 to 79)	−13 (1.6 to 22), <0.001
3.9–7.8 mmol/L	34 (19 to 47)	45 (33 to 56)	−16 (2.3 to 22.4), <0.001
<3.9 mmol/L	1.4 (0.0 to 5.6)	1.5 (0.0 to 2.1)	−0.5 (−3.8 to 1.4), 0.071
<3.3 mmol/L	0.1 (0.0 to 1.7)	0.0 (0.0 to 0.7)	−0.0 (−1.5 to 0.2), 0.11
<2.8 mmol/L	0.0 (0.0 to 0.6)	0.0 (0.0 to 0.3)	−0.0 (−0.3 to 0.0), 0.46
>7.8 mmol/L	63 (48 to 79)	55 (42 to 64)	−14 (−28 to −1), 0.0015
>10.0 mmol/L	38 (19 to 56)	30 (18 to 40)	−12 (−21 to 0), 0.0054
>13.9 mmol/L	10 (2 to 21)	8 (2 to 13)	−3 (−10 to 2), 0.032
Mean glucose level (mmol/L)	9.2 (8.1 to 10.8)	8.8 (7.9 to 9.3)	−0.8 (−2.1 to 0.4), 0.013
SD of glucose (mmol/L)	3.2 (2.6 to 3.8)	3.0 (2.3 to 3.7)	−0.3 (−0.6 to 0.4), 0.25
CV of glucose (%)	34.4 (30.2 to 36.2)	33.3 (29.1 to 38.7)	−1.5 (−4.2 to 7.5), 0.82
Basal insulin (U/6-h nighttime)	5.4 (4.1 to 7.7)	6.6 (5.0 to 9.4)	−1.3 (0.3 to 2.1), <0.001
Bolus insulin (U/6-h nighttime)	0.8 (0.3 to 1.6)	0.4 (0.1 to 1.1)	−0.5 (−0.7 to 0.0), 0.047

Data are median (IQR).

^a^ Automated insulin delivery minus sensor-augmented pump therapy.

Bolus insulin delivery was lower with automated insulin delivery compared with sensor-augmented pump therapy, whereas basal insulin delivery was higher with automated insulin delivery (Table 2). During the day, median bolus insulin delivery was 21.3 U/18 h with automated insulin delivery compared with 22.6 U/18 h with sensor-augmented pump therapy (*P* = 0.064; Table 3), whereas basal insulin delivery was 19.4 U/18 h with automated insulin delivery compared with 17.7 U/18 h with sensor-augmented pump therapy (*P* = 0.0012; Table 3). During the night, bolus insulin delivery was 0.4 U/6 h with automated insulin delivery compared with 0.8 U/6 h with sensor-augmented pump therapy (*P* = 0.047; Table 3), whereas basal insulin delivery was 6.6 U/6 h with automated insulin delivery compared with 5.4 U/6 h with sensor-augmented pump therapy (*P* < 0.001; Table 3). Between-day variability in insulin delivery was higher with automated insulin delivery compared with sensor-augmented pump therapy during the night (30% vs. 25%; *P* = 0.0043), but not during the day (16% vs. 15%; *P* = 0.56).

All participants responded to the questionnaire. Participants reported higher treatment satisfaction with automated insulin delivery. The average score of the Diabetes Treatment Satisfaction Questionnaire^22^ was 26.4 (5.6) at baseline, 26.9 (5.7) after sensor-augmented pump therapy, and 28.2 (5.5) after automated insulin delivery (Supplementary Appendix).

## Discussion

Several randomized trials tested dosing algorithms for automated insulin delivery systems, comparing its efficacy to sensor-augmented pump therapy, in outpatient unsupervised settings with no remote monitoring.^7–12,14^ Here, we present a relatively short study over 12 days that assesses our dosing algorithm in outpatient unsupervised settings. The algorithm was a less aggressive version than our previous algorithm,^18–20^ to emphasize safety in our first outpatient study. In the relatively well-controlled population included in our study, automated insulin delivery system increased time spent in the target glucose range and reduced time spent in hypoglycemia, and the benefits were observed during the day and night. Moreover, treatment satisfaction was increased with the automated insulin delivery system, in line with patients' expectations regarding the prospective use of automated insulin delivery systems.^23^

The two large studies of the University of Cambridge and the University of Virginia algorithms included a review of data and adjustments of therapy parameters after 2 weeks of system usage.^8,10^ Moreover, it has been reported that patients transitioning to the 670G system usually adjust their insulin-to-carbohydrate ratios to be more aggressive by 15%–20%.^24^ Due to the relatively short duration of our study, we did not incorporate a therapy optimization step during the automated insulin delivery intervention. Longer studies with our system may allow for such adjustments, which may further impact glucose control.

Other algorithms were tested in outpatient settings, but with the use of remote monitoring^25^ or without a control arm.^13,26^ Remote monitoring and remote interventions at times of impending hypoglycemia could bias the study by underestimating hypoglycemia outcomes (hypoglycemia outcomes will be the result of the automated insulin delivery system plus the remote monitoring interventions). Automated insulin delivery studies without a control arm may demonstrate feasibility and safety; however, it is difficult to draw conclusions on the system's effectiveness, as the outcomes may be partially driven by the baseline characteristics of the study participants or simply by participation in the study (the trial effect). More randomized-controlled trials in free-living settings with no remote monitoring are needed.

Unlike other algorithms,^10,14^ our algorithm does not administer automatic boluses outside mealtimes. Algorithms that deliver automatic boluses in addition to modulating background basal rates are not necessarily more aggressive than algorithms that only modulate background basal rates. Increasing basal rates by several folds over a short period of time can have the same effect as delivering a bolus (e.g., 6 U/h over 10 min delivers 1 U), and algorithms only modulating background basal rates have been shown to improve glucose control compared with sensor-augmented pump therapy^7–9^ as much as algorithms delivering automatic boluses.^10,14^

In our study, no difference was observed between outcomes of week 1 and week 2, suggesting that the benefits of automated insulin delivery are immediate. This is also in line with results from longer studies over 3 to 6 months from other groups,^7,10^ where benefits were observed after only 2 weeks. However, showing statistically significant benefits compared with control after 2 weeks does not necessarily mean that the numerical estimates of the outcomes are accurate and representative of longer term use. Correlation analysis indicates that a duration of 4 weeks might be needed to reliably obtain representative numerical estimates of time-in-target range and mean glucose outcomes,^27^ although using a correlation analysis to obtain this duration has been recently questioned.^28^

Despite the significant improvement in time-in-target range with automated insulin delivery, the numerical reduction in mean glucose level of 0.4 mmol/L did not reach statistical significance. This might have been related to that the study was not powered to detect a difference in mean glucose level. Also, since the mean glucose level was reduced significantly during the night but not during the day (Table 3), more aggressive prandial dosing might have led to a bigger numerical improvement in the overall mean glucose level, which might have in turn led to statistical significance.

Limitations of our study include its relatively short duration over 12 days, the lack of allocation blinding, and that participants did not use the same pump in both arms. Strengths include the randomized-controlled design and the unsupervised settings. Our study confirms findings that automated insulin delivery improves glucose control compared with sensor-augmented pump therapy. Using our system in future research projects, such as those assessing a simplified carbohydrate counting strategy,^29,30^ is now warranted.

## Supplementary Material

Supplemental data

## Supplementary Appendix

Clinical Study Protocol

Diabetes Treatment Satisfaction Questionnaire Outcomes
