# Supplementary material for: Comparison Between Closed-Loop Insulin Delivery System (the Artificial Pancreas) and Sensor-Augmented Pump Therapy: A Randomized-Controlled Crossover Trial
Source: Diabetes Technol Ther. 2021 Feb 25;23(3):168–74. doi: 10.1089/dia.2020.0365 (PMC7906861; doi:10.1089/dia.2020.0365)
Supplement: Supplemental data [file Supp_Data.zip › Supplementary Data.pdf]

Clinical Study Protocol

CLASS14 – Closed-Loop Assessment Study

"An open-label, randomized, two-way, crossover trial to assess the efficacy of single-hormone closed-loop system and sensor-augmented pump therapy to regulate day-and-night glucose levels for 12 days in free-living outpatient conditions in adults with type 1 diabetes"

Principal investigator:

Dr Rémi Rabasa-Lhoret, MD, PhD
Director, Research Platform on Obesity, Metabolism and Diabetes
Full time professor, Faculty of Medicine, Nutrition department, Université de Montréal
Institut de recherches cliniques de Montréal
110, avenue des Pins Ouest
Montreal (Quebec) Canada H2W 1R7

Co-investigators:

Dr Ahmad Haidar, PhD
Assistant professor
Biomedical Engineering
McGill University
3775, University
Montreal (Quebec) Canada H3A 2B4

Dr Laurent Legault, MD
Assistant professor
Division of Endocrinology
Department of Pediatrics
Montreal Children's Hospital
1001, Decarie boulevard
Montreal (Quebec) Canada H4A 3J1

Sponsor:
Dr Rémi Rabasa-Lhoret, MD, PhD
Director, Research Platform on Obesity, Metabolism and Diabetes
Institut de recherches cliniques de Montréal
110, avenue des Pins Ouest
Montreal (Quebec) Canada H2W 1R7

Investigation site:
Institut de recherches cliniques de Montréal
110, avenue des Pins Ouest
Montreal (Quebec) Canada H2W 1R7

Centre for Innovative Medicine at McGill University Health Centre Research Institute
1001, Decarie boulevard
Montreal (Quebec) Canada H4A 3J1

Funded by: National Institutes of Health
Protocol no: 2018-938
Version 10: June ^{4th}, 2019

Table of Contents

1 Background and Rationale 4
2 Identification of Investigational Device and Drugs 5
3 Risks and Benefits for Participants 7
3.1 Anticipated clinical benefits 7
3.2 Anticipated adverse device effects 7
3.3 Residual risks associated with study devices 8
3.4 Risks associated with participation in the study 8
3.5 Steps to control and mitigate the risks 9
3.6 Risk-to-benefit rationale 10
4 Trial Objectives 10
5 Hypothesis 11
6 Study Design 11
6.1 Trial Design 11
6.2 Study Population 11
6.2.1 Inclusion criteria 11
6.2.2 Exclusion criteria 11
6.3 Study Interventions 12
6.3.1 Single-hormone closed-loop strategy 12
6.3.2 Sensor-augmented pump therapy (SAPT) 12
7 Study Procedures and Visit Schedule 13
7.1 Recruitment 13
7.2 Visit Schedules 13
7.2.1 Admission Visit (Visit 1) 13
7.2.2 Study interventions (Visit 2 and 3) 14
7.3 Randomization 15
8 Statistical Analysis 15
8.1 Study Endpoints 15
8.1.1 Primary endpoint 15
8.1.2 Secondary endpoints 15
8.2 Sample Size and Power Calculations 16
8.3 Level of Significance 16

8.4 Statistical Tests 16
8.4.1 Primary endpoint analysis 16
8.4.2 Secondary endpoint analysis 17
9 Test Patients 17
10 Monitoring Plan 18
11 Adverse events, Adverse device effects and Device deficiencies 19
11.1 Definitions 19
11.2 Responsibilities of the Principal Investigator 20
11.3 Reporting of SAE, SADE and USADE 20
11.4 Reporting Device Deficiencies 21
12 Suspension or Premature Termination of the Study 21
12.1 Suspension of the study 22
12.2 Premature termination of the study 22
13 Ethical and Legal Consideration 22
13.1 Good Clinical Practice 22
13.2 Delegation of Investigators Duties 22
13.3 Participant Information and Informed Consent: 23
13.4 Confidentiality 23
13.5 Approval of the Clinical Study Protocol and Amendments 23
13.6 Record Retention 24
14 Publication Policy 24
15 References 24

1 Background and Rationale

The closed-loop system (CLS) is composed of 3 components: a glucose sensor (to measure glucose levels), an infusion pump (to infuse insulin) and a dosing algorithm to control insulin delivery based on real-time glucose concentrations to maintain glucose levels in an optimal target range.

The first studies conducted with an automated CLS were targeting overnight glucose control [1-4]. Many studies are now assessing the effectiveness of CLS to regulate day-and-night glucose levels in outpatient settings [5-10]. Leelarathna et al. [7] conducted a 7-day multicenter randomized crossover study in 17 adults. Glucose levels were in the target range for 75% of the time with CLS compared to 62% with sensor-augmented pump therapy (SAP). Bally et al. [5] compared the efficacy of CLS and conventional insulin pump therapy to regulate day-and-night glucose levels in adults with T1D. The proportion of time when glucose levels was in target range (3.9-10.0 mmol/L) was 10.5 percentage points higher when the CLS was used compared to insulin pump therapy. The CLS also reduced the percentage of time spent in hypoglycemia. Tauschmann et al. [9] evaluated the efficacy of single-hormone CLS and SAP to regulate glucose levels for 21 days in adolescents with T1D. Percentage of time of glucose levels spent in the target range and above target range as well as mean glucose level was improved with the CLS compared to SAP. *Overall, these studies suggest that use of CLS is associated with an increase in time spent in the target range and also a reduction in mean blood glucose and/or time spent in hypoglycemia.*

We developed an automated single-hormone (insulin) closed-loop system using the Tandem t:slim insulin pump and Dexcom G5 glucose sensor. We plan to use this system in a multicenter randomized study conducted in outpatient conditions in 100 adolescents and adults with T1D. Before initiating this trial, we believe that it is important to test this closed-loop system over a shorter period of time. Therefore, we aim to conduct a randomized trial comparing single-hormone automated CLS (insulin) and sensor-augmented pump therapy (SAP) in regulating day-and-night glucose levels for 12 days in 36 adults with T1D.

2 Identification of Investigational Device and Drugs

The following devices will be used in this study:

1. The **Tandem t:slim Insulin Pump** is a fully integrated insulin delivery system developed by Tandem (San Diego, California, USA). It includes 3 components: an external insulin pump connected to an infusion set (tubing and catheter). It contains an insulin reservoir of 3.0 mL corresponding to a maximum of 300U capacity and can deliver both the basal rate and a bolus (e.g. meal insulin dose) in a fast, flexible and discreet way.
2. The **Dexcom G5 Glucose Sensor** will be used as a Continuous Glucose Monitoring System (CGMS). This device has been developed by Dexcom (San Diego, California, USA). The system is composed of a small electrochemical sensor placed just under the skin, a transmitter connected to the sensor, and a Smartphone (or a Dexcom receiver) on which glucose values are shown. The sensor probe is positioned at a 45° angle under the skin. It is placed with the help of an applicator barrel and can be used for up to seven days. Using amperometry, the sensor utilizes a working electrode coated with a sensing element (Wired Enzyme) that converts glucose concentration to electrical current. The transmitter snaps into the sensor and wirelessly sends, every 5 minutes, glucose information to the Smartphone (or to the Dexcom receiver). Customizable alarms can be set in the Smartphone (or in the Dexcom receiver) to inform the patient about potential (e.g. rapid decline in glucose levels) or actual hypoglycemia or hyperglycemia (specific threshold). Once installed, the glucose sensor needs a 2-hour period of initialization before providing any continuous glucose data. According to manufacturer's recommendations, the sensor has to be calibrated 2 hours after the insertion and at least twice a day thereafter with a capillary blood glucose value.
3. The **dosing algorithm** is integrated in a LG Google Nexus Smartphone. The algorithm is initialized with daily insulin requirements and insulin-to-carbohydrate ratios. The dosing algorithm will calculate the insulin doses to administer every 10 minutes.
4. A **glucose meter** will be used to calibrate the glucose sensor. The participant will be asked to use the same glucose meter throughout the study to calibrate the glucose sensor.

The following drugs will be used in this study:

1. Insulin: The two fast-acting insulin analogs used in the study are:

a. Novorapid (Aspart) by Novo Nordisk
b. Humalog (Lispro) by Eli Lilly and Company

If participant’s usual insulin analog is different than above, participants will be switched to Aspart or Lispro for the duration of the study.

A device-tracking log will allow us to trace which devices were given to a participant for the duration of their participation. This device-tracking log will include:

- Serial number of insulin pumps
- Serial number of continuous glucose monitoring systems
- Identification number of LG Google Nexus phones

All participants will have their glucose levels regulated for 12 days with:
1) single-hormone CLS;
2) sensor-augmented pump therapy.

Details on the intervention with the CLS are given in Section 6.3. This study is conducted in outpatient settings in adults with type 1 diabetes. Detailed inclusion and exclusion criteria are enumerated in Section 6.2. Training on all study devices will be given to participants. Only participants who show competence in using the study devices will be allowed to participate in the study.

Participation in this study involves:

- The installation of two glucose sensors (replacement after 6 days) for each study intervention and the sensor calibrations;
- The installation of a catheter for the infusion of insulin. Participants will be asked to change the catheter every 2-3 days, as per routine practice. In some situations (e.g. hyperglycemia), participants might have to change the catheter more often;
- The completion of a journal for hypoglycemic and hyperglycemic episodes as well as catheter and reservoir changes.

3 Risks and Benefits for Participants

3.1 Anticipated clinical benefits

The purpose of this trial is to compare the efficacy of automated single-hormone CLS with sensor-augmented pump therapy in regulating glucose levels over a 12-day period in free-living outpatient conditions in adults with T1D.

In the short-term, based on the evaluation of sensor glucose values obtained during the study, participants will have the opportunity to optimize their treatment in a way that may improve how they treat their diabetes. This includes resolving catheter issues as well as improving choice of basal insulin levels and insulin boluses.

In the long-term, it is hoped that the CLS will help patients to achieve recommended glucose targets more efficiently, safely, and simply.

3.2 Anticipated adverse device effects

As with any insulin pump device, the patient could encounter *catheter problems* (e.g. disconnection, kinking, leaking, etc.) that usually lead to hyperglycemia. We will review with patients how to handle hyperglycemic episodes and provide them with a ketone meter, additional catheters and insulin syringes to ensure optimal management. We will review with patients how to appropriately insert the catheter. As patients will be asked to change their catheter each 2-3 days we do not anticipate local infection problem which are mostly observed for continuous wear beyond 3 days.

The Dexcom glucose monitoring system includes alarms (e.g. for hypoglycemia and hyperglycemia); additional alarms have been incorporated in the CLS (e.g. lost connection). *Alarm fatigue* has been described in the medical literature [11]. Patients will be allowed to use their own glycemic targets with the obligation of having the lower target above 3.1 mmol/L. A team member will be available to help patients to choose these settings based on their medical history (e.g. hypoglycemia unawareness, diabetes control), their experience if they are already using a glucose sensor, etc. For patients who are not already using a continuous glucose monitoring system, the first days using this system can be associated with anxiety related to the

discovery of unknown and unperceived episodes of hypoglycemia and hyperglycemia. This aspect will be addressed during the run-in period allowing insulin treatment to be optimized.

Over and/or under-reading with glucose sensors is well described in the literature, which could lead to hypoglycemia or hyperglycemia. Most recent sensor generations have minimized these problems. In addition, patients will be asked to undertake optimal calibrations measures. Finally, the patients will be reminded about situations in which over and/or under-reading can occur (e.g. prolonged physical activity).

3.3 Residual risks associated with study devices

The combination of bench testing, previous experience with CLS, patient selection, pre-trial education, optimal care measures during the trial (e.g. optimal glucose sensor calibration), and constant support should minimize residual risk.

In case of lost connection between the pump and the phone or between the transmitter and the phone, the CLS will automatically switch to open-loop delivery of the usual insulin basal rate used by the patient. An alarm will warn the patients that they are not anymore in closed-loop mode. In such a case, it is assumed that patients will face a risk of hypoglycemia or hyperglycemia similar to what they face using their usual diabetes treatment.

3.4 Risks associated with participation in the study

Hypoglycemia: Even though the efficacy of the CLS to reduce the occurrence of hypoglycemia has been shown, it is always possible that participants might experience hypoglycemic episodes during the 12-day intervention. Participants will be recommended to treat hypoglycemia according to guidelines: consume 15g of carbohydrates if capillary glucose is below 4 mmol/L or if they have symptoms of hypoglycemia. They will be recommended to measure capillary glucose 15 minutes later and repeat treatment if glucose levels are still below 4 mmol/L or if they still have symptoms of hypoglycemia. If they treat 5 or more hypoglycemic episodes (capillary glucose < 4.0 mmol/L) in one day, they will be asked to contact the research team. If they experience a severe hypoglycemia event (third party assistance is necessary to treat hypoglycemia), they will be asked to immediately end the study intervention and to contact the research team. Patients will be reminded about risk factors for hypoglycemia, symptoms and

treatment. In addition, patients will be asked to always have carbohydrates available and if they do not own an intact glucagon kit, one will be provided and they will be asked to keep it with them during the entire trial.

Hyperglycemia: Even though the efficacy of the CLS in reducing the occurrence of hyperglycemia has been shown, it is always possible that participants might experience hyperglycemic episodes during the 12-day intervention. Participants will be instructed to measure blood ketones levels when capillary glucose is above 16 mmol/L for at least one hour. If blood ketones are above 1.5 mmol/L, it will be important to take necessary actions to correct the situation. These measures include: an insulin correction bolus, proper hydration, changing the insulin infusion set and/or insulin reservoir and eventually delivering a bolus of insulin with a syringe or insulin pen. Participants will be asked to monitor glucose levels and blood ketones levels and to have regular contact with the research team upon resolution of hyperglycemia and ketonemia. If the situation is not resolved within 6 hours, participants will be asked to prematurely end the intervention. The procedure for ketone management is described in detail in Annex A.

Blood sampling: Blood sampling will be performed by qualified personnel at the admission visit. This procedure may still cause mild discomfort. Bruises (blue) or more infrequently dizziness may occur.

Glucose sensor, catheter for continuous infusion of insulin: Study procedures are not expected to increase known risk related to insertion sites. There is a risk of infection at the points of insertion for the catheters and sensors as well as possible irritation. It is possible to feel a slight discomfort when the catheter or the sensor is inserted.

3.5 Steps to control and mitigate the risks

Algorithm to control glucose values: In our previous studies [12-19], the algorithm used for single-hormone CLS has shown promising results with reduced hypoglycemic and hyperglycemic risk as compared to conventional pump therapy.

Automated device: The closed-loop device includes multiple safety features including alarms and ability to switch to open-loop mode. Extensive bench testing has been undertaken to ensure

that the automated device is safe. Prior to the main clinical trial, up to 6 patients will have tested the automated CLS devices at the research facility for 24 hours under the supervision of the research team.

Per-protocol: Patients will receive education about the device. Only patients showing adequate understanding device use and compliance to study procedures will be allowed to continue their participation in the trial. Regular contact with patients (e.g. phone contact on the 1st evening) and data review after 6 days will ensure careful monitoring. The study team will be available 24/7 for any technical or medical issues. In addition, for the 1st day of CLS arm, patients will be asked to activate the exercise mode of the CLS before driving for more than 10 minutes. In case of problems, we will ask patients to allow us to access the study app data and we will undertake a rapid revision of glucose profiles.

3.6 Risk-to-benefit rationale

The most common side effect of all available treatments with insulin is hypoglycemia. The risk of hypoglycemia with the CLS is anticipated to be equal to or lower than sensor-augmented pump therapy, the best available treatment at the moment. Recent data strongly suggest that using a continuous glucose monitoring device reduces the hypoglycemic risk even in patients at high risk of hypoglycemia [20].

The CLS has also shown its ability to improve the time for which glucose levels are in the target range. It is thus expected that the time glucose levels spend in the target range with the CLS will be the same as or better than the time they would spend in the target range with sensor-augmented pump therapy.

Overall, the risk of both hypoglycemia and hyperglycemia should not be higher and could be lower during trial participation.

4 Trial Objectives

To compare the efficacy of automated single-hormone CLS with sensor-augmented pump therapy in improving percentage of time of glucose levels spent in the target range for a 12-day period in free-living outpatient conditions in adults with T1D.

5 Hypothesis

Automated single-hormone CLS will increase time spent between 3.9 and 10.0 mmol/L in adults with T1D compared to sensor-augmented pump therapy.

6 Study Design

6.1 Trial Design

CLASS14 (Closed-loop Assessment Study) is an open-label, randomized, two-way, cross-over study comparing single-hormone (insulin) CLS and sensor-augmented pump therapy in regulating glucose levels for a 12-day period in adults with T1D.

6.2 Study Population

The trial aims to enroll adult subjects with T1D using insulin pump therapy.

6.2.1 Inclusion criteria

To be eligible for the study, all subjects must meet the following criteria:

1. Males and females ≥ 18 years of old.
2. Clinical diagnosis of type 1 diabetes for at least one year.
   The diagnosis of type 1 diabetes is based on the investigator’s judgment; C peptide level and antibody determinations are not needed.
3. The subject will have been on insulin pump therapy for at least 3 months.
4. HbA1c ≤ 12%.

6.2.2 Exclusion criteria

Subjects who meet any of the following criteria are not eligible for the study:

1. Clinically significant nephropathy, neuropathy or retinopathy as judged by the investigator
2. Recent (< 6 months) acute macrovascular event e.g. acute coronary syndrome or cardiac surgery
3. Warfarin chronic treatment if INR monitoring cannot be evaluated (can increase the risk of bleeding)

4. Chronic use of acetaminophen. Acetaminophen may interfere with glucose sensor readings
5. Pregnancy (ongoing or current attempt to become pregnant)
6. Breastfeeding
7. No nearby party for assistance if needed
8. Plans to go abroad or travel at more than 2 hours distance from Montreal during the trial period
9. Severe hypoglycemic episode within two weeks of screening or during the run-in period
10. Severe hyperglycemic episode requiring hospitalization in the last 3 months
11. Current use of glucocorticoid medication (except low stable dose and inhaled steroids)
12. Known or suspected allergy to the trial products
13. Other serious medical illness likely to interfere with study participation or with the ability to complete the trial by the judgment of the investigator
14. Anticipation of a significant change in exercise regimen between admission and end of the trial (i.e. starting or stopping an organized sport)

6.3 Study Interventions

6.3.1 Single-hormone closed-loop strategy

During single-hormone closed-loop interventions, variable subcutaneous insulin infusion will be used to regulate glucose levels. Aspart or Lispro will be infused using a subcutaneous infusion pump (Tandem t:slim, Tandem). Every 10 minutes, the glucose levels as measured by the sensor (Dexcom G5, Dexcom) will be transferred automatically to a LG Google Nexus Smartphone that the algorithm is running on, which will calculate the recommended doses and will send them wirelessly to the infusion pump. The dosing recommendations are based on a predictive algorithm [21]. Predictive algorithms have been successfully used in closed-loop studies, see for example [22] and [23] for studies in children and adults. The algorithm used in this project is an evolution of the ones used in our previously completed CLASS01 [14], CLASS02 [24], CLASS03 [16], CLASS04 [18], CLASS05 [13], CLASS06 [19], CLASS07 [17], CLASS08 [15], and CLASS10 [12] studies.

6.3.2 Sensor-augmented pump therapy (SAPT)

During control interventions, participants will use sensor-augmented pump therapy to regulate glucose levels. Aspart or Lispro will be used.

7 Study Procedures and Visit Schedule

7.1 Recruitment

Subjects will be recruited at the Institut de recherches cliniques de Montréal (IRCM) and Centre for Innovative Medicine at McGill University Health Centre Research Institute. Potential subjects (T1D subjects under pump therapy) will be approached by the research team. Subjects showing interest in participation will have the study fully explained to them, will be given a copy of the consent form, and will be offered the opportunity to ask questions. Interested subjects that meet basic eligibility criteria will be scheduled for the admission visit (Visit 1).

7.2 Visit Schedules

7.2.1 Admission Visit (Visit 1)

At the admission visit, the following procedures will be taken:

- Inclusion and exclusion criteria will be assessed.
- Subjects will be asked to sign the consent form.
- A medical visit will be taken to establish medical history (i.e. recent severe hypo- and hyperglycemia events, micro- and macrovascular complications, comorbidities, and precise list of all medications).
- A1c level if no recent (< 1 month) result is available.
- Weight, height and waist circumference will be obtained.
- Records of the previous 3 days of insulin therapy will be obtained (total daily dose, carbohydrate to insulin ratios, basal rates).
- Training on the use of the pump and sensor will be provided.
- Subjects will be asked to complete the Diabetes Treatment Satisfaction questionnaire [25].

After the admission visit, the participants will have a run-in period of 12 days using the study sensor. During the run-in period, participants will use their insulin pump. After 6 days (± 2 days) and 10 days (± 2 days) of the run-in period, an experienced member of the team (nutritionist or nurse) will review the pump and sensor data and will contact study participants to optimize their treatment parameters (insulin to carbohydrate ratios, basal rates, etc) if required. The objective

of this contact is to make obvious therapeutic adjustments (i.e. to address unperceived nocturnal hypoglycemia).

7.2.2 Study interventions (Visit 2 and 3)

For interventions with the single-hormone CLS, the sensor will be installed at least one day before the start of the intervention by the participants. On the first day of an intervention with the single-hormone CLS, participants will be admitted to the clinical research facility anytime between 8:00 am and 11:30 am. The study insulin pump will be installed and participants will be trained on the closed-loop system, and that includes training on connection and disconnection of the system and meal boluses (a user guide will also be given to participants). Sensor alarm thresholds will be determined by participants. Competency using study devices will be assessed by a team member. Only participants demonstrating competency using study devices will be allowed to continue to the home study phase. Participants will be discharged in the afternoon and advised to continue with study intervention at home for the next 12 days.

On the first day of an intervention with the single-hormone closed-loop system, participants will be asked to activate the exercise mode of the CLS for the following situations: driving for more than 10 minutes, manual work requiring great attention and exercise at moderate or high intensity for more than 30 minutes. On the subsequent days, participants will be asked to measure their capillary glucose level before driving, undertaking manual work requiring great attention or performing an exercise at moderate or high intensity for more than 30 minutes. If the capillary glucose level is below 6.0 mmol/L, participants will need to eat or drink at least 15g of carbohydrates. For all these situations, participants will be asked to measure their capillary glucose level every hour. Participants will be advised to have carbohydrates with them at all times.

For intervention with sensor-augmented pump therapy, the sensor will be installed at least one day before the start of the intervention by the participants. Participants will use their own insulin pump.

For Closed-Loop intervention, participants will be contacted on the first evening to ensure that they have not encountered any unexpected events or technical problems. Additional phone calls will be planned on day 3 and day 9. For the SAPT intervention, participants will be contacted on

Day 3 (± 2 days) and Day 9 (± 2 days) as they are already contacted on Day 1 for the start of control intervention.

For both interventions, participants will be asked to record hypoglycemia episodes, calibrations, catheter and sensor changes and hyperglycemic episodes with ketones in a diary provided to them. After each intervention, participants will be asked to complete the diabetes treatment satisfaction questionnaire. This questionnaire will be used to evaluate the impact of, and the satisfaction with study devices.

The first intervention visit will be maximum30 days after the run-in period. Intervention visits should be separated by 4-40 days. We will have 24-hour technical support available during all interventions.

The duration of each intervention will be 12 days.

7.3 Randomization

Each study participant will be assigned a unique anonymous identification number, which will be used throughout the study. A block-balanced randomization will be used to determine the order of the interventions. Randomization envelopes will be opened at the admission visit.

8 Statistical Analysis

8.1 Study Endpoints

8.1.1 Primary endpoint

Percentage of time of glucose levels spent in the target range (3.9-10.0 mmol/L).

8.1.2 Secondary endpoints

1. Percentage of time of glucose levels spent *a.* between 3.9 and 7.8 mmol/L; *b.* below 3.9 mmol/L; *c.* below 3.3 mmol/L; *d.* below 2.8 mmol/L; *e*. above 10.0 mmol/L; *f.* above 13.9 mmol/L; *g.* above 16.7 mmol/L.
2. Percentage of time (00:00-6:00) of glucose levels spent *a.* below 3.9 mmol/L; *b.* between 3.9 and 7.8 mmol/L; *c.* between 3.9 and 10.0 mmol/L; *d.* below 3.3 mmol/L; *e.* below 2.8 mmol/L; *f*. above 10.0 mmol/L; *g.* above 13.9 mmol/L; h. above 16.7 mmol/L.

3. Mean glucose levels.
4. Fasting sensor glucose levels.
5. Standard deviation of glucose levels and insulin delivery.
6. Coefficient of variance of glucose levels and insulin delivery.
7. Between-day variability in glucose levels and insulin delivery.
8. Total insulin delivery.
9. Number of hypoglycemic events less than 3.1 mmol/L (>20 minutes).
10. Number of nights with hypoglycemic events less than 3.1 mmol/L (>20 minutes).
11. Number of days with hypoglycemic events less than 3.1 mmol/L (>20 minutes).
12. Total number of hours and percentage of time of sensor availability.
13. Time (hours) between failures due to sensor unavailability.
14. Time (hours) between failures due to pump connectivity.
15. Number of days with at least one technical problem.
16. Number of hours and percentage of time when patients switched back to sensor-augmented pump therapy.
17. Number of hours and percentage of time when the CLS was automatically switched to sensor-augmented pump therapy.
18. Number of calls and patients calling for technical issues related to CLS.

8.2 Sample Size and Power Calculations

The required sample size to obtain a power of 80% to detect a difference of at least 15% in the percentage of time of glucose levels spent in the target range between with single-hormone CLS and sensor-augmented pump therapy, under a paired design, assuming a common SD of 22% in each arm, and a two-sided Type I error (α-level) of 5%, is 36 patients. Calculations were done using SAS 9.4 power procedure.

8.3 Level of Significance

5% significance threshold will be used to declare statistical significance.

8.4 Statistical Tests

8.4.1 Primary endpoint analysis

The primary endpoint for this study is percentage of time of glucose levels spent in the target range (3.9-10.0 mmol/L), and will be compared between single-hormone CLS vs. sensor-

augmented pump therapy. To estimate the difference between the two interventions on the primary endpoint, we will use a linear mixed effect model (LMEM) with the intervention, treatment sequence, period, and starting glucose entered as fixed effect covariates and subject nested within sequence as random effect. The model is suited for repeated observations, i.e., adjusts for patient-level intra-correlation. Residual values will be examined for normality. Because time in target data have positive values that can follow a non-normal distribution, non-parametric bootstrap procedures will be used to estimate parameters and their 95% confidence interval as a sensitivity analysis.

8.4.2 Secondary endpoint analysis

A similar statistical strategy employed for the analysis of the primary endpoint will be used to compare treatment effects for all continuous secondary outcomes. Significance level is set at 5%. Sensitivity analysis, including non-parametric bootstrap method and zero-inflated model will be adopted following the distribution of the data.

Hypoglycemic episode and other count variables will be compared among intervention using a linear mixed effect model with Poisson family (or negative binomial), adjusted for intervention, sequence, and period as fixed effect and individuals as random effect.

9 Test Patients

Up to six test patients will be recruited to assess the engineering performance of automated single-hormone CLS in regulating glucose levels in free-living inpatient conditions. Participants will stay at the Centre for Innovative Medicine at McGill University Health Centre Research Institute or at Institut de recherches cliniques de Montréal under the supervision of a nurse or a research staff with an MD on site or on call. No admission visits is required (data obtained from usual medical file), but may be conducted, for these patients. Participants will be admitted at the clinical research facility between 7:30 am and 11:00 am and will stay at the research facility for 24 hours. A glucose sensor will be inserted by the participant. Participants will be trained on the closed-loop system, and that includes training on connection and disconnection of the system and meal boluses (a user guide will also be given to participants). Participants will have the freedom to choose their day schedule. Participants will freely choose their meals from a large choice of pre-prepared frozen meals. Fresh fruits and vegetables, cheese, yogurt, beverages and bread will also be available at any time. Applicable endpoints detailed in Section 8 will be

calculated after the all test patients have completed the in-patient intervention. The data of the test patients will not be used in the statistical analysis of the main trial.

10 Monitoring Plan

A research assistant at IRCM not implicated in this research project will be in charge of monitoring data.

A first monitoring will be done after the first participant completes the study. Afterwards, monitoring will occur after each five patients complete the study.

The monitoring plan includes verifying that:

- The study is conducted according to the study protocol as well as according to Good Clinical Practice guidelines and local regulation;
- Protocol deviations have been documented and reported to the research ethics board;
- Study devices are being used according to the study protocol;
- Signed and dated informed consent forms have been obtained from each participant at the point of enrollment or before any study procedures are undertaken;
- Case report forms and other study documents are accurate, complete, up to date, stored and maintained appropriately;
- Appropriate corrections, additions or deletions are made to case report forms, dated, explained if necessary and initiated by the person who did the corrections, additions or deletions;
- All serious adverse events and device deficiencies have been reported to the research ethics board;
- All other documents related to the study and correspondence are maintained in the study binder;
- Participant’s withdrawal has been documented.

All monitoring activities will be documented in a written report kept in the study binder. This report will include:

- The date of the monitoring visit;
- The name of the monitor;
- A summary of what the monitor reviewed;
- Significant findings and protocol deviations.

A Data Safety Monitoring Board will be composed of 3 researchers in the field of diabetes and with knowledge about CLS. The role of the Data Safety Monitoring Board will be to periodically review and evaluate the study data for participant safety, study conduct and progress, and to make recommendations concerning the continuation, modification, or termination of the trial. The first DSMB meeting will be planned after the first three patients complete the study. Additional DSMB meeting will be planned in the case of an SAE (e.g. severe hypoglycemia) to ensure rapid revision (within less than 1 week) and provide advice to the clinical team.

11 Adverse events, Adverse device effects and Device deficiencies

11.1 Definitions

Adverse event (AE): any untoward medical occurrence, unintended disease or injury, or untoward clinical signs in participants whether or not related to the study devices.

Adverse device effect (ADE): adverse event related to the use of a study device. This definition includes adverse events resulting from insufficient or inadequate instructions for use, or any malfunction of the study device. This definition also includes any event resulting from use error or from intentional misuse of the study device.

Device deficiency: inadequacy of the study device with respect to its reliability, safety or performance.

Serious adverse event (SAE): adverse event that a) led to death; b) led to serious deterioration in the health of the subject, that either resulted in 1) a life-threatening illness or injury, or 2) a persistent or significant disability/incapacity, or 3) in-patient or prolonged hospitalization, or 4)

medical or surgical intervention to prevent life-threatening illness or injury or persistent or significant disability/incapacity.

Serious adverse device effect (SADE): adverse device effect that has resulted in any of the consequences characteristic of a serious adverse event.

Unanticipated serious adverse device effect (USADE): serious adverse device effect which by its nature, incidence, severity or outcome has not been identified in the current version of the risk analysis report.

11.2 Responsibilities of the Principal Investigator

The principal investigator is responsible for:

- The classification of AE and evaluation of their seriousness and relationship to the study device.
- Reviewing all device deficiencies and determine and document in writing whether they could have led to a SADE.
- Reporting to the research ethics board all SAE and device deficiencies within 30 days of becoming aware of the problem.
- Reporting to the research ethics board and regulatory authorities all SAE, SADE and USADE within 10 days of becoming aware of the problem.
- Reporting all relevant safety information to the Data Safety Monitoring Board.
- Ensuring that the research ethics board and the regulatory authorities are informed of significant new information about the study, and in case of SADE and device deficiencies that could have led to SADE, determine whether the risk analysis needs to be updated and assess whether corrective or preventive action is required.

11.3 Reporting of SAE, SADE and USADE

When reporting an SAE, SADE or USADE, the following information will be presented:

- Intensity: the intensity of an adverse event can be classified as mild, moderate or severe.
    - **Mild**: no or transient symptoms, no interference with the subject's daily activities.
    - **Moderate**: marked symptoms, moderate interference with the subject's daily activities.

- **Severe**: considerable interference with the subject's daily activities; unacceptable.

- Relationship to study procedures/devices: based on the principal investigator's judgement, the relationship to study procedures/devices can be probable, possible or unlikely.
  - **Probable**: good reason and sufficient documentation to assume a causal relationship.
  - **Possible**: a causal relationship is conceivable and cannot be dismissed.
  - **Unlikely**: the event is most likely related to aetiology other than the trial product.
- Final outcome:
  - Recovered/resolved: the participant has fully recovered.
  - Recovering/resolving: the condition is improving and the participant is expected to recover from the event.
  - Recovered/resolved with sequel: the participant has recovered from the condition, but with lasting effect due to a disease, injury, treatment or procedure.
  - Not recovered/not resolved: the condition of the participant has not improved and the symptoms are unchanged.
  - Fatal: this term is only applicable if the participant died from a condition related to the reported AE.
  - Unknown: this term is only applicable if it is not possible to follow up with the participant.

11.4 Reporting Device Deficiencies

When reporting a device deficiency, the following information will be presented:

- Description of the deficiency as well as the circumstances in which the deficiency happened.
- Measures to correct the deficiency.
- Report if the deficiency induced an adverse event.

12 Suspension or Premature Termination of the Study

The principal investigator, the REB, the DSMB or Health Canada may decide to suspend or stop the trial at any time.

If a trial is suspended or prematurely terminated, the principal investigator must inform the participants as well as the REB and Health Canada and provide a detailed written explanation.
If in the course of the study, the benefit-risk analysis changes, the new evaluation must be provided to the REB and Health Canada.
If the study is suspended, it can then only be resumed after obtaining a new written approval by REB and Health Canada.

12.1 Suspension of the study

The study can be suspended by the DSMB based on data or SAE review.

12.2 Premature termination of the study

The study will be prematurely ended 1) if the DSMB establish that the risk-to-benefit ratio of the trial is not acceptable, 2) if three or more patients encounter an SAE related to the study devices with single-hormone CLS or 3) if a death occurs with single-hormone CLS and it is established that the cause of death is related to the CLS.

13 Ethical and Legal Consideration

13.1 Good Clinical Practice

This study is to be conducted according to globally accepted standards of good clinical practice (as defined in the ICH E6 Guideline for Good Clinical Practice, 1 May 1996), in agreement with the Declaration of Helsinki and in keeping with local regulations.

13.2 Delegation of Investigators Duties

The investigator should ensure that all persons assisting with the trial are adequately qualified, informed about the protocol, any amendments to the protocol, the study treatments, and their trial-related duties and functions.

The investigator should maintain a list of sub-investigators and other appropriately qualified persons to whom he or she has delegated significant trial-related duties.

13.3 Participant Information and Informed Consent:

After reading the relevant documents, the participant must give consent in writing. This consent must be confirmed by the personally dated signature of the participant and by the personally dated signature of the person conducting the informed consent discussions.

A copy of the signed consent documents must be given to the participant. The original signed consent documents will be retained by the investigator.

The investigator will not undertake any measures specifically required only for the clinical study until valid consent has been obtained.

It is suggested that the investigator inform the participant's primary physician about the participant's participation in the trial, if the participant has a primary physician other than the study investigator.

13.4 Confidentiality

Participant names will be kept in strictest confidence. Participants will be identified by their participant identification numbers which does not contain date of birth or initials. Study data stored on a computer will be stored in accordance with local data protection laws.

The investigator will maintain a personal participant identification list (participant numbers with the corresponding participant names) to enable records to be identified. Participants' identifiers and contact details will be stored locally under strict security.

13.5 Approval of the Clinical Study Protocol and Amendments

Before the start of the study, the clinical study protocol, informed consent document, and any other appropriate documents will be submitted to the IEC/IRB with a cover letter or a form listing the documents submitted, their dates of issue, and the site for which approval is sought.

Before the first participant is enrolled in the study, formal written approval from the IEC/IRB must be obtained and all ethical and legal requirements must be met.

The IEC/IRB must be informed of all subsequent protocol amendments and administrative changes, in accordance with local legal requirements.

The investigator must keep a record of all communication with the IEC/IRB.

13.6 Record Retention

All study records must be kept according to ICH guidelines.

14 Publication Policy

The principal investigator commits to communicating, and otherwise making available for public disclosure, results of this study. Public disclosure includes publication of a paper in a scientific journal, abstract submission with a poster or oral presentation at a scientific meeting, or disclosure by other means. No confidential information will be disclosed.

15 References

1. Hovorka, R., et al., *Overnight closed-loop insulin delivery in young people with type 1 diabetes: a free-living, randomized clinical trial.* Diabetes Care, 2014. 37(5): p. 1204-11.
2. Ly, T.T., et al., *Overnight glucose control with an automated, unified safety system in children and adolescents with type 1 diabetes at diabetes camp.* Diabetes Care, 2014. 37(8): p. 2310-6.
3. Nimri, R., et al., *MD-Logic overnight control for 6 weeks of home use in patients with type 1 diabetes: randomized crossover trial.* Diabetes Care, 2014. 37(11): p. 3025-32.
4. Thabit, H., et al., *Home use of closed-loop insulin delivery for overnight glucose control in adults with type 1 diabetes: a 4-week, multicentre, randomised crossover study.* Lancet Diabetes Endocrinol, 2014. 2(9): p. 701-9.
5. Bally, L., et al., *Day-and-night glycaemic control with closed-loop insulin delivery versus conventional insulin pump therapy in free-living adults with well controlled type 1 diabetes: an open-label, randomised, crossover study.* Lancet Diabetes Endocrinol, 2017. 5(4): p. 261-270.
6. El-Khatib, F.H., et al., *Home use of a bihormonal bionic pancreas versus insulin pump therapy in adults with type 1 diabetes: a multicentre randomised crossover trial.* Lancet, 2017. 389(10067): p. 369-380.
7. Leelarathna, L., et al., *Day and night home closed-loop insulin delivery in adults with type 1 diabetes: three-center randomized crossover study.* Diabetes Care, 2014. 37(7): p. 1931-7.
8. Russell, S.J., et al., *Day and night glycaemic control with a bionic pancreas versus conventional insulin pump therapy in preadolescent children with type 1 diabetes: a randomised crossover trial.* Lancet Diabetes Endocrinol, 2016. 4(3): p. 233-43.
9. Tauschmann, M., et al., *Home Use of Day-and-Night Hybrid Closed-Loop Insulin Delivery in Suboptimally Controlled Adolescents With Type 1 Diabetes: A 3-Week, Free-Living, Randomized Crossover Trial.* Diabetes Care, 2016. 39(11): p. 2019-2025.
10. Tauschmann, M., et al., *Day-and-Night Hybrid Closed-Loop Insulin Delivery in Adolescents With Type 1 Diabetes: A Free-Living, Randomized Clinical Trial.* Diabetes Care, 2016. 39(7): p. 1168-74.

11. Tansey, M., et al., *Satisfaction with continuous glucose monitoring in adults and youths with Type 1 diabetes.* Diabet Med, 2011. 28(9): p. 1118-22.
12. Gingras, V., et al., *A Simplified Semiquantitative Meal Bolus Strategy Combined with Single- and Dual-Hormone Closed-Loop Delivery in Patients with Type 1 Diabetes: A Pilot Study.* Diabetes Technol Ther, 2016. 18(8): p. 464-71.
13. Gingras, V., et al., *Efficacy of dual-hormone artificial pancreas to alleviate the carbohydrate-counting burden of type 1 diabetes: A randomized crossover trial.* Diabetes Metab, 2016. 42(1): p. 47-54.
14. Haidar, A., et al., *Glucose-responsive insulin and glucagon delivery (dual-hormone artificial pancreas) in adults with type 1 diabetes: a randomized crossover controlled trial.* CMAJ, 2013. 185(4): p. 297-305.
15. Haidar, A., et al., *Outpatient overnight glucose control with dual-hormone artificial pancreas, single-hormone artificial pancreas, or conventional insulin pump therapy in children and adolescents with type 1 diabetes: an open-label, randomised controlled trial.* Lancet Diabetes Endocrinol, 2015. 3(8): p. 595-604.
16. Haidar, A., et al., *Comparison of dual-hormone artificial pancreas, single-hormone artificial pancreas, and conventional insulin pump therapy for glycaemic control in patients with type 1 diabetes: an open-label randomised controlled crossover trial.* Lancet Diabetes Endocrinol, 2015. 3(1): p. 17-26.
17. Haidar, A., et al., *Outpatient 60-hour day-and-night glucose control with dual-hormone artificial pancreas, single-hormone artificial pancreas, or sensor-augmented pump therapy in adults with type 1 diabetes: An open-label, randomised, crossover, controlled trial.* Diabetes Obes Metab, 2017. 19(5): p. 713-720.
18. Haidar, A., et al., *Single- and Dual-Hormone Artificial Pancreas for Overnight Glucose Control in Type 1 Diabetes.* J Clin Endocrinol Metab, 2016. 101(1): p. 214-23.
19. Taleb, N., et al., *Efficacy of single-hormone and dual-hormone artificial pancreas during continuous and interval exercise in adult patients with type 1 diabetes: randomised controlled crossover trial.* Diabetologia, 2016. 59(12): p. 2561-2571.
20. van Beers, C., et al., *Continuous glucose monitoring for patients with type 1 diabetes and impaired awareness of hypoglycaemia (IN CONTROL): a randomised, open-label, crossover trial.* Lancet Diabetes Endocrinol, 2016. Epub ahead of print.
21. Hovorka, R., *Continuous glucose monitoring and closed-loop systems.* Diabetic Medicine, 2006. 23(1): p. 1-12.
22. El-Khatib, F.H., et al., *A Bihormonal Closed-Loop Artificial Pancreas for Type 1 Diabetes.* Science Translational Medicine, 2010. 2(27): p. -.
23. Hovorka, R., et al., *Manual closed-loop insulin delivery in children and adolescents with type 1 diabetes: a phase 2 randomised crossover trial.* Lancet, 2010. 375(9716): p. 743-751.
24. Haidar, A., et al., *Post-breakfast closed-loop glucose control is improved when accompanied with carbohydrate-matching bolus compared to weight-dependent bolus.* Diabetes Metab, 2014. 40(3): p. 211-4.
25. Juvenile Diabetes Research Foundation Continuous Glucose Monitoring Study, G., *Validation of measures of satisfaction with and impact of continuous and conventional glucose monitoring.* Diabetes Technol Ther, 2010. 12(9): p. 679-84.

Annex A. Ketone Management Procedure

Participants will be asked to measure their capillary ketones when blood glucose levels are above **16 mmol/L for a period longer than an hour** and/or when they experience symptoms of hyperglycemia (increased urination, significant thirst, fatigue, blurred vision, stomach aches, headaches, nausea, vomiting …).

If blood ketones levels are:

Between 0.0 and 0.6 mmol/L, instruct participant to:

- Drink sugar-free and caffeine-free fluids.
- Check the cartridge, catheter and insertion site.
- Avoid skipping meal and have a minimal amount of carbohydrate (> 30g) in such meal.
- Use the iMAP or participant personal insulin pump to calculate a correction bolus. Administer correction bolus via the pump.
- Monitor signs and symptoms of hyperglycemia (excessive thirst, urination, abdominal pain, nausea, weakness, etc…) and ketones.
- Monitor blood glucose and ketone levels 2 hours after correction bolus is administered.
- If hyperglycemia persists (> 16 mmol/L) and/or blood ketone levels increase 2 hours post correction, change infusion set and follow ketone management procedure.

Between 0.6 and 1.5 mmol/L, instruct participant to:

- Contact their study nurse
- Drink sugar-free and caffeine-free fluids (aim 200-250 ml every 30-60 minutes)
- Use bolus calculator on insulin pump or iMAP to calculate correction bolus. Administer the bolus via syringe or insulin pen. (If on CLS, the participant should disconnect the study pump from their body and deliver the same bolus through the system, so it accounts for the insulin given via the syringe, and proper IOB is calculated)

**Participant should be encouraged to eat a small amount of carbs (15-45g), there should be a bolus administered for any carb intake.*

- Change the pump cartridge, tubing and infusion set.
- Verify if cannula seems kinked or blocked, and if there is blood at the old insertion site. Check if their insulin was properly stored (as per supplier recommendations). This information should be charted in participant file by the study nurse.
- Monitor signs and symptoms of hyperglycemia (excessive thirst, urination, abdominal pain, nausea, weakness, etc…) and ketones
- Monitor blood glucose and blood ketones levels 2 hours after correction bolus is administered
- If hyperglycemia persists or blood ketone levels remain positive (one or both values have not decreased from initial values), notify study physician and keep supporting participant by following the ketone management procedure.

Above 1.5 mmol/L, instruct participant to:

- Contact their study nurse (study physician should be notified immediately for ketone values above 3.0 mmol/L).
- Drink sugar-free and caffeine-free fluids (aim at 200-250 ml every 30-60 minutes)
- Use bolus calculator on insulin pump or iMAP to calculate correction bolus. Multiply **correction bolus** by 1.5. (Ex correction bolus recommendation = 2U, Multiply by 1.5 = 3U). Administer the bolus via syringe or insulin pen. If on CLS, the participant should disconnect the study pump from their body and deliver the same bolus through the system, so it accounts for the insulin given via the syringe, and proper IOB (insulin on board) is calculated.
- **Participant should be encouraged to eat a small amount of carbs (15-45g), there should be a bolus administered for any carb intake. It's important that only the correction part of the bolus is multiplied.*
- Change the cartridge, tubing and infusion set.
- Verify if cannula seems kinked or blocked, and if there is blood at the old insertion site. Check if their insulin was properly stored (as per supplier recommendations). This information should be charted in participant file by the study nurse.

- Monitor signs and symptoms of hyperglycemia (excessive thirst, urination, abdominal pain, nausea, weakness, etc…) and ketones
- Monitor blood glucose and blood ketones levels 2 hours after correction bolus is administered
- If hyperglycemia persists or blood ketone levels remain positive (one or both values have not decreased from initial values), notify study physician and keep supporting participant by following the ketone management procedure.

In the event of positive blood ketones (above 0,6 mmol/L), the participant will only return to pump or iMAP pump therapy once ketones are below 0,6 mmol/L.

Participants will record all blood ketone levels measured and infusion set changes in the Study Participant Journal. The reasons for infusion set changes are documented in the journal (ex: planned vs unplanned).

Reporting

All positive blood ketones events (> 0.6 mmol/L) will be documented as Adverse Events (AE) in the participant chart. Should the local ERB require submission for this type of event, AE will be submitted to ERB for review. Should the AE be classified as serious by study physician (hospitalization required, etc…), the event will be documented as SAE and will be reported to ERB, DSMB and regulatory authorities.
