# Supplementary figures and images for: Comparison Between Closed-Loop Insulin Delivery System (the Artificial Pancreas) and Sensor-Augmented Pump Therapy: A Randomized-Controlled Crossover Trial
Source: Diabetes Technol Ther. 2021 Feb 25;23(3):168–74. doi: 10.1089/dia.2020.0365 (PMC7906861; doi:10.1089/dia.2020.0365)

# Diabetes Treatment Satisfaction Questionnaire Outcomes


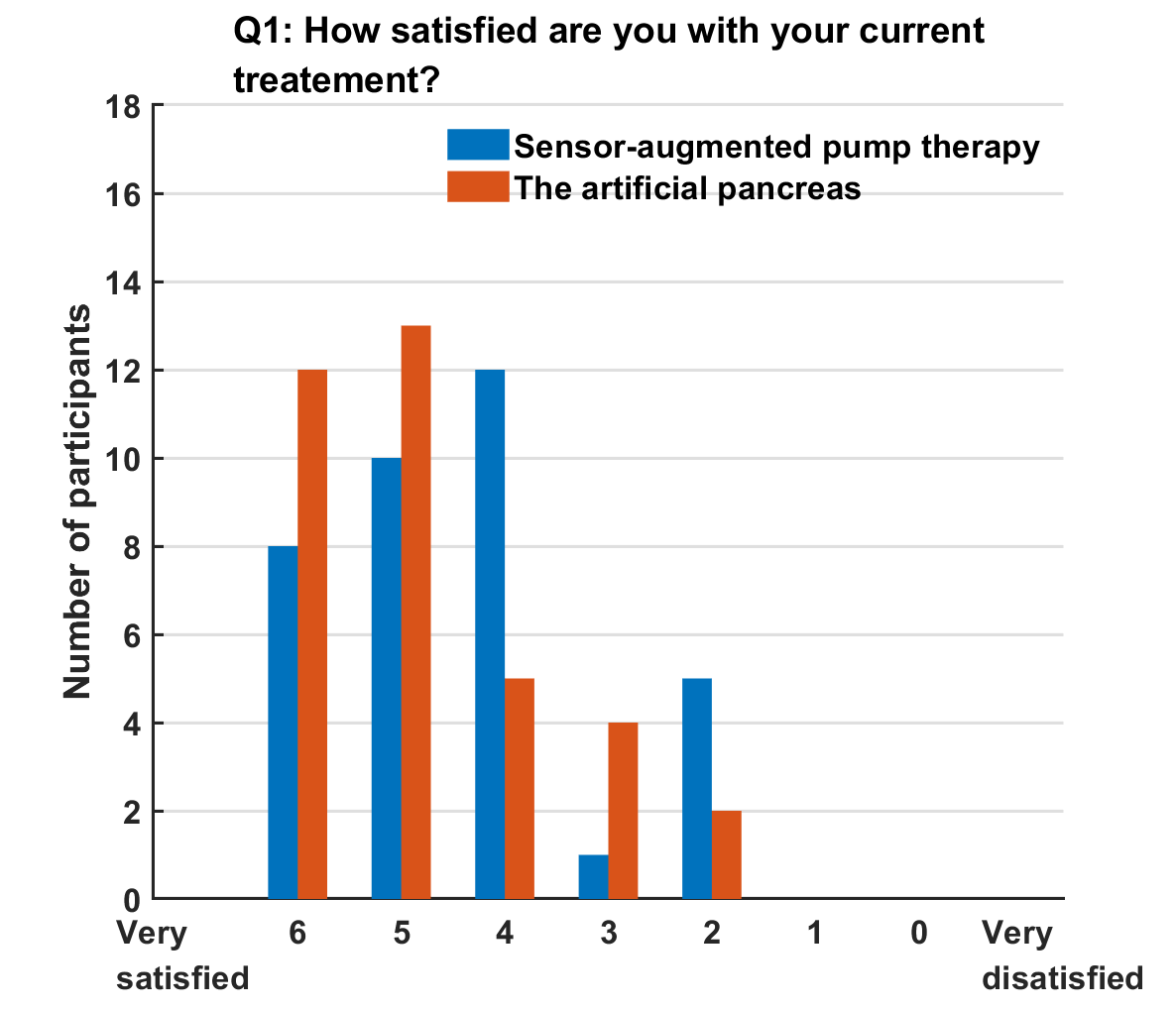


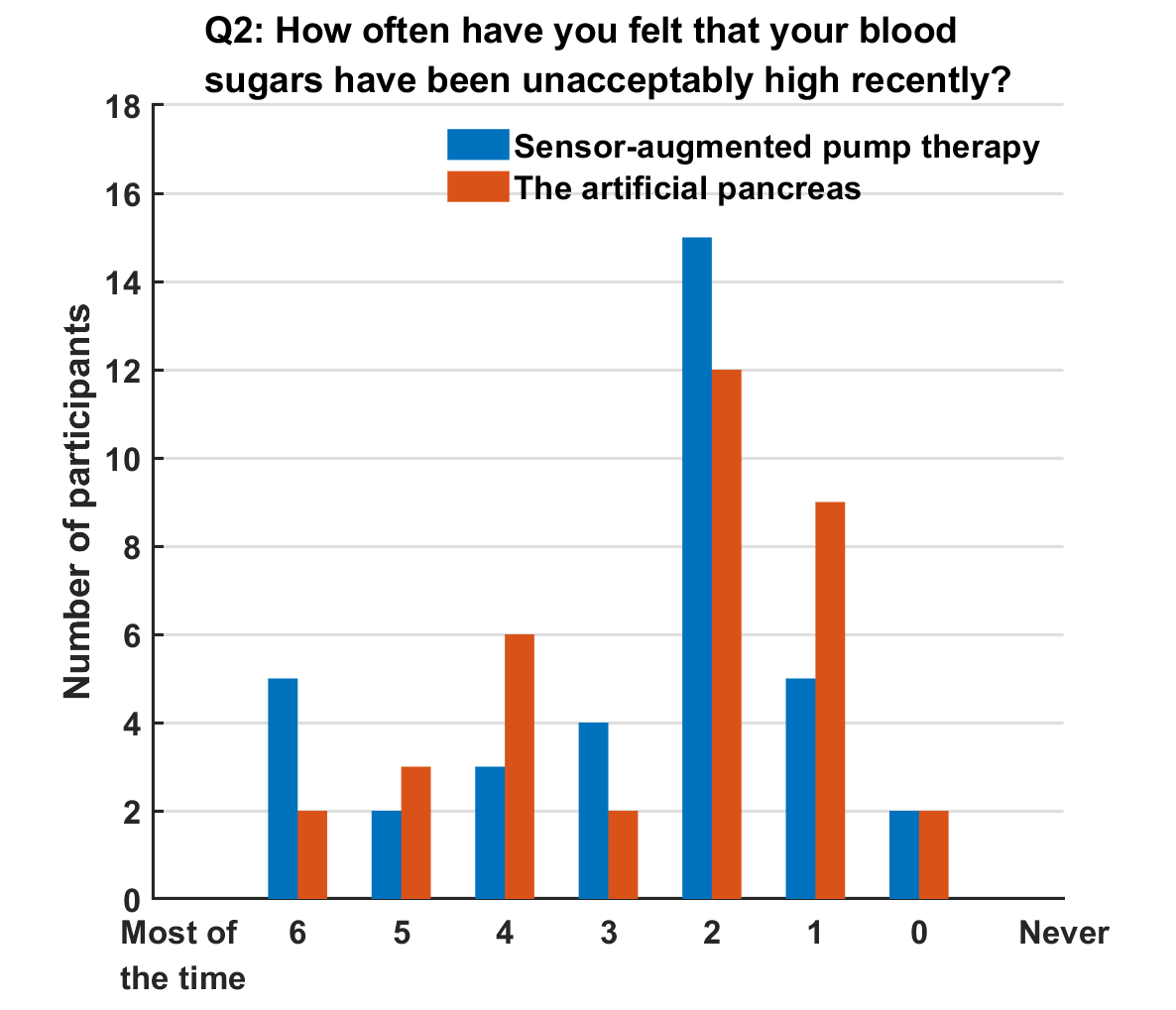


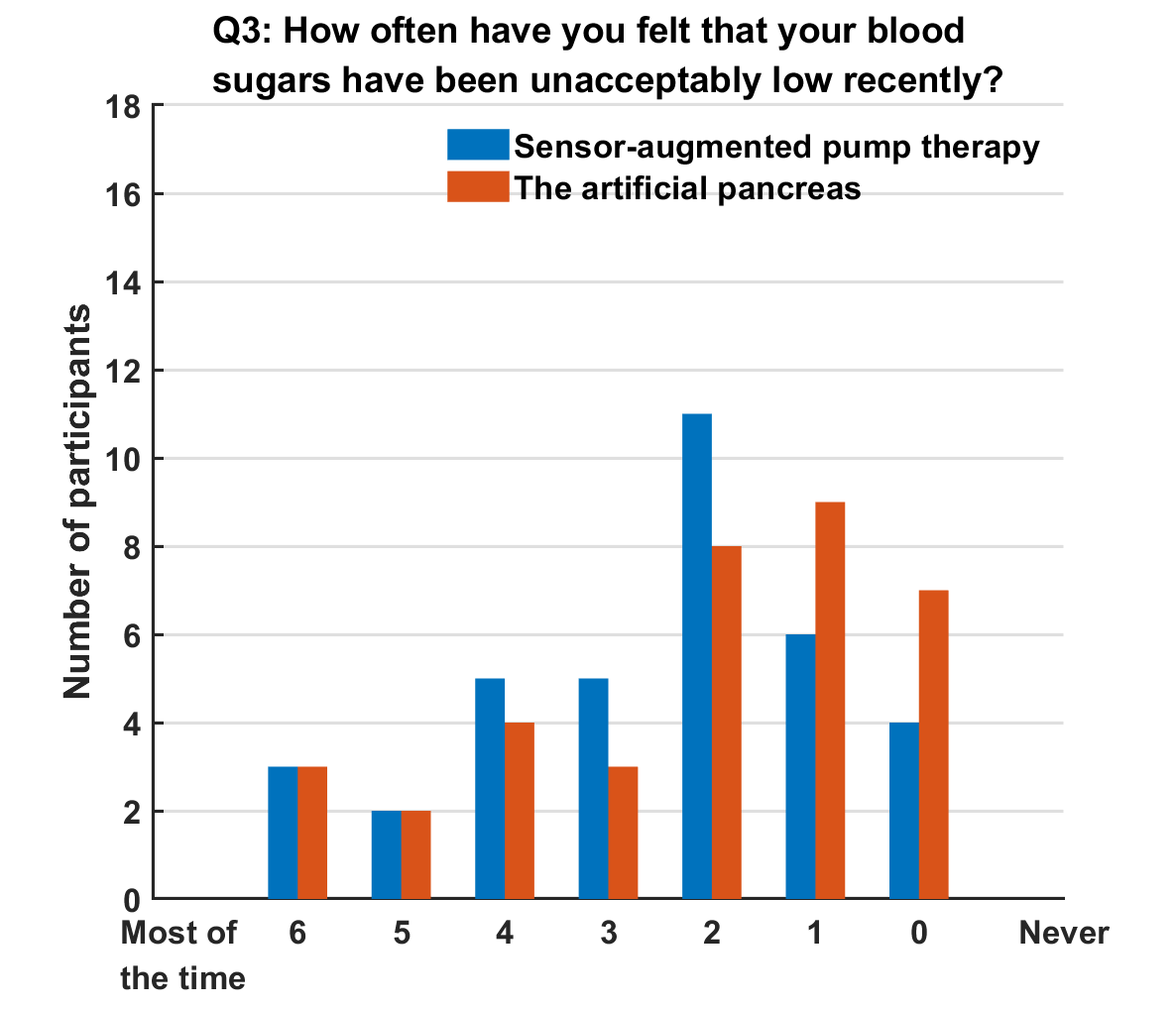


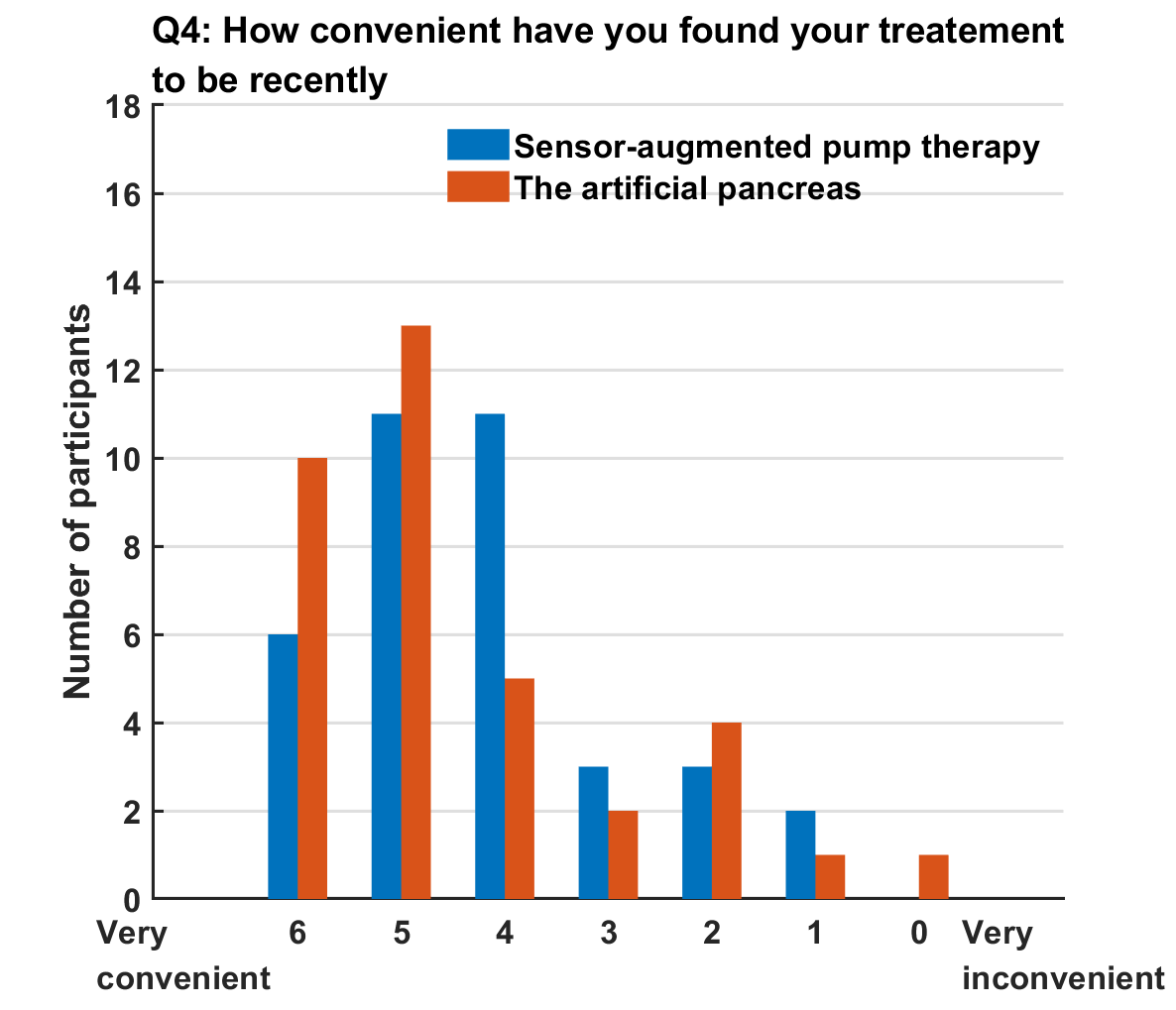


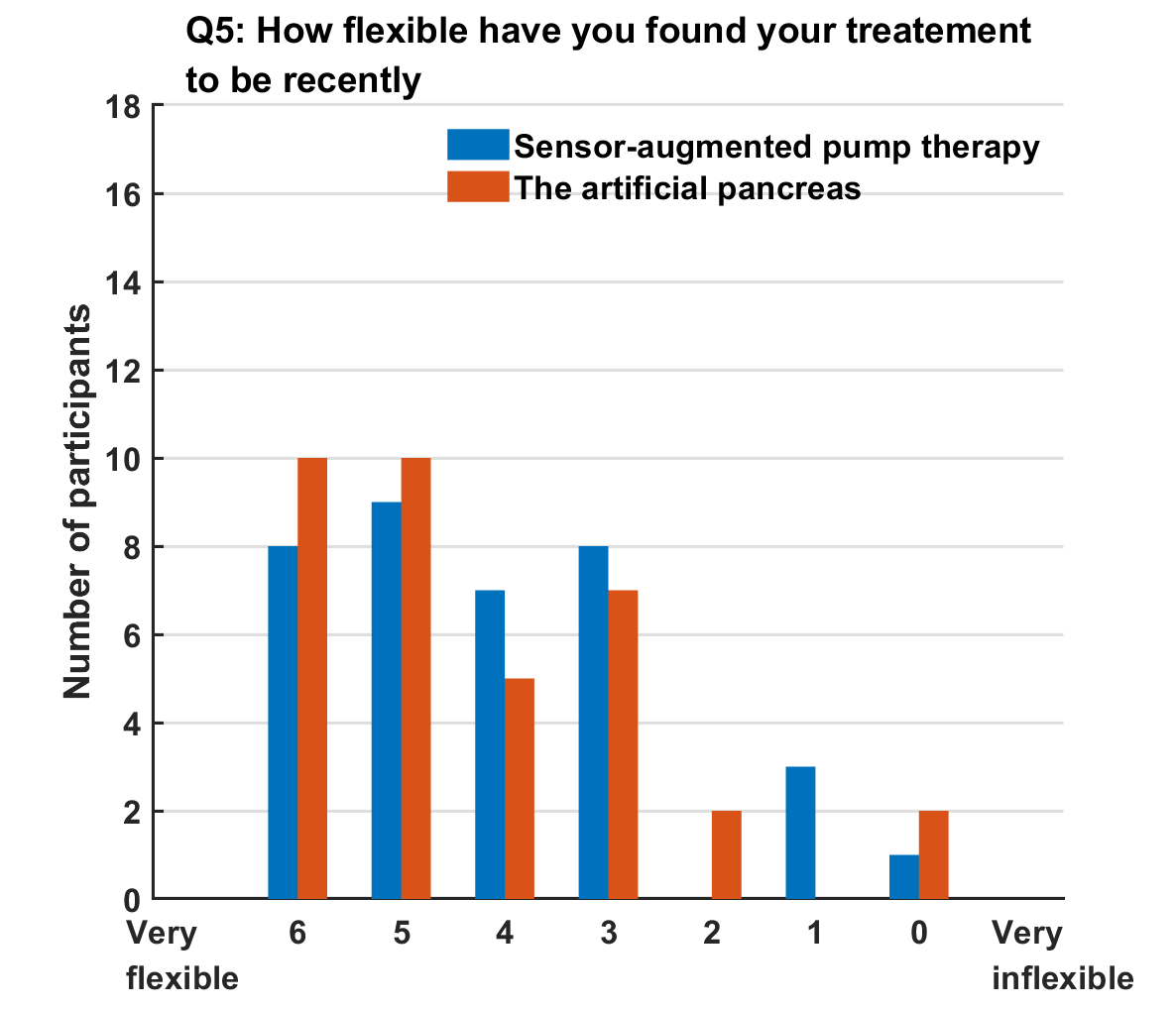


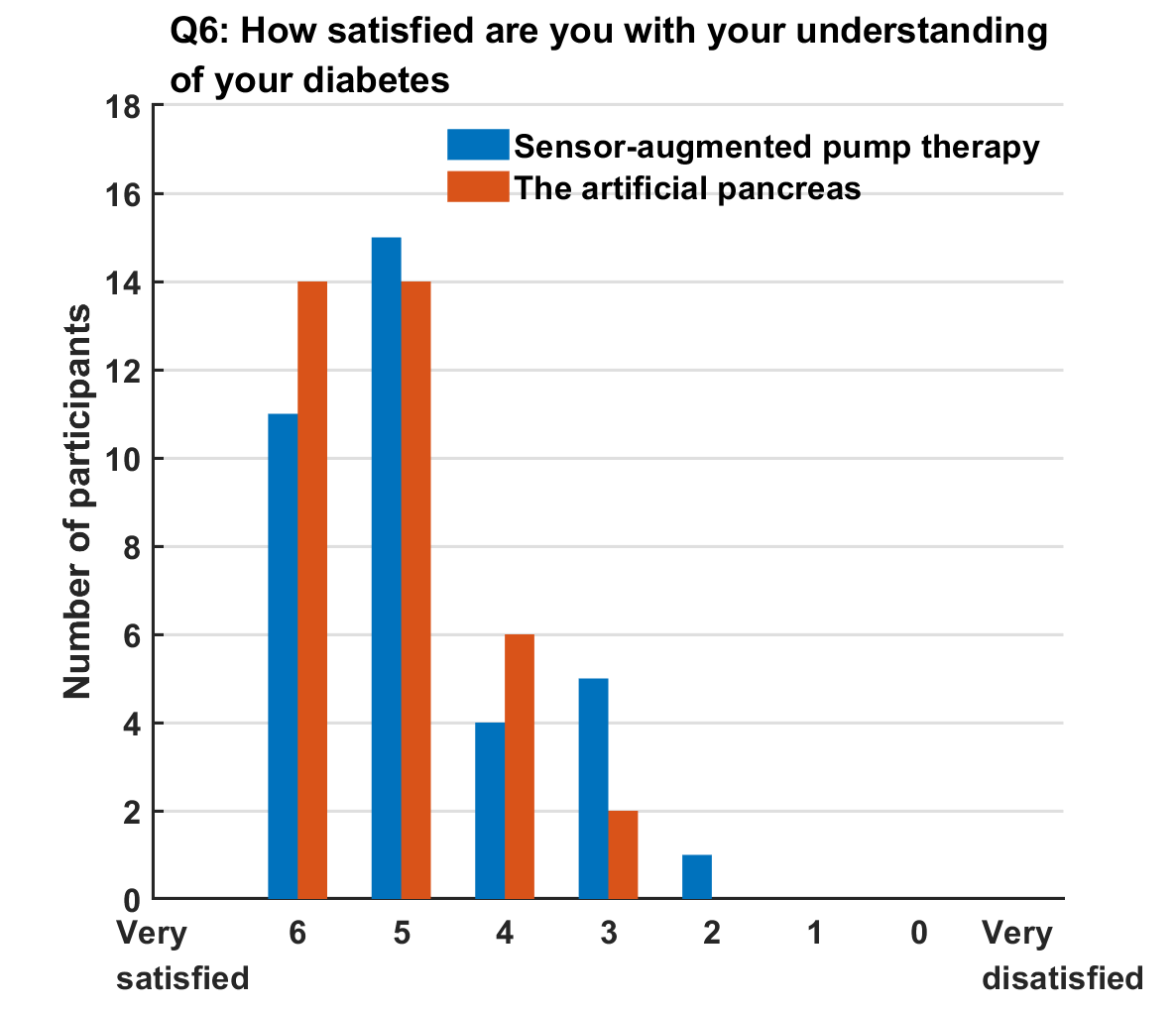


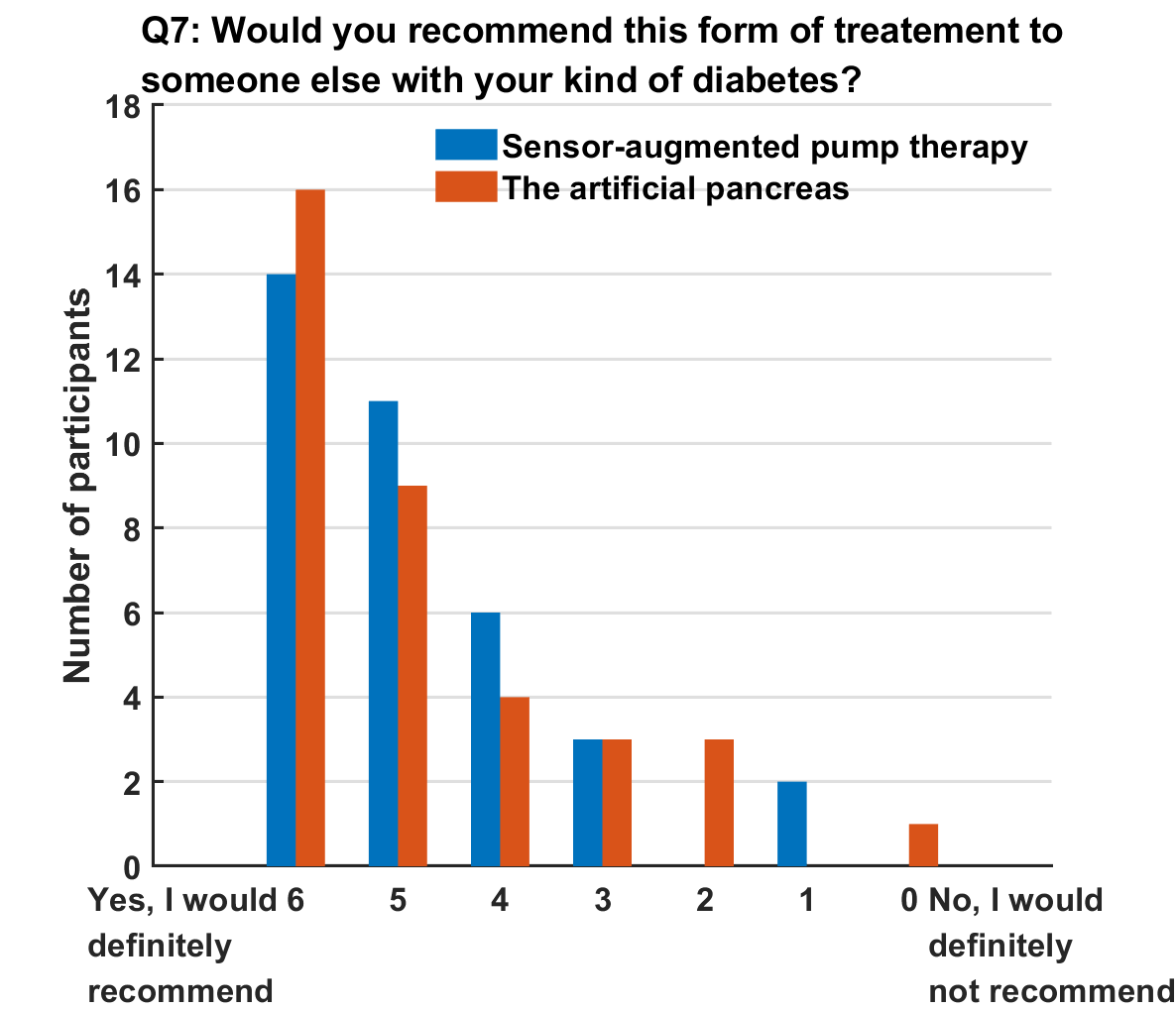


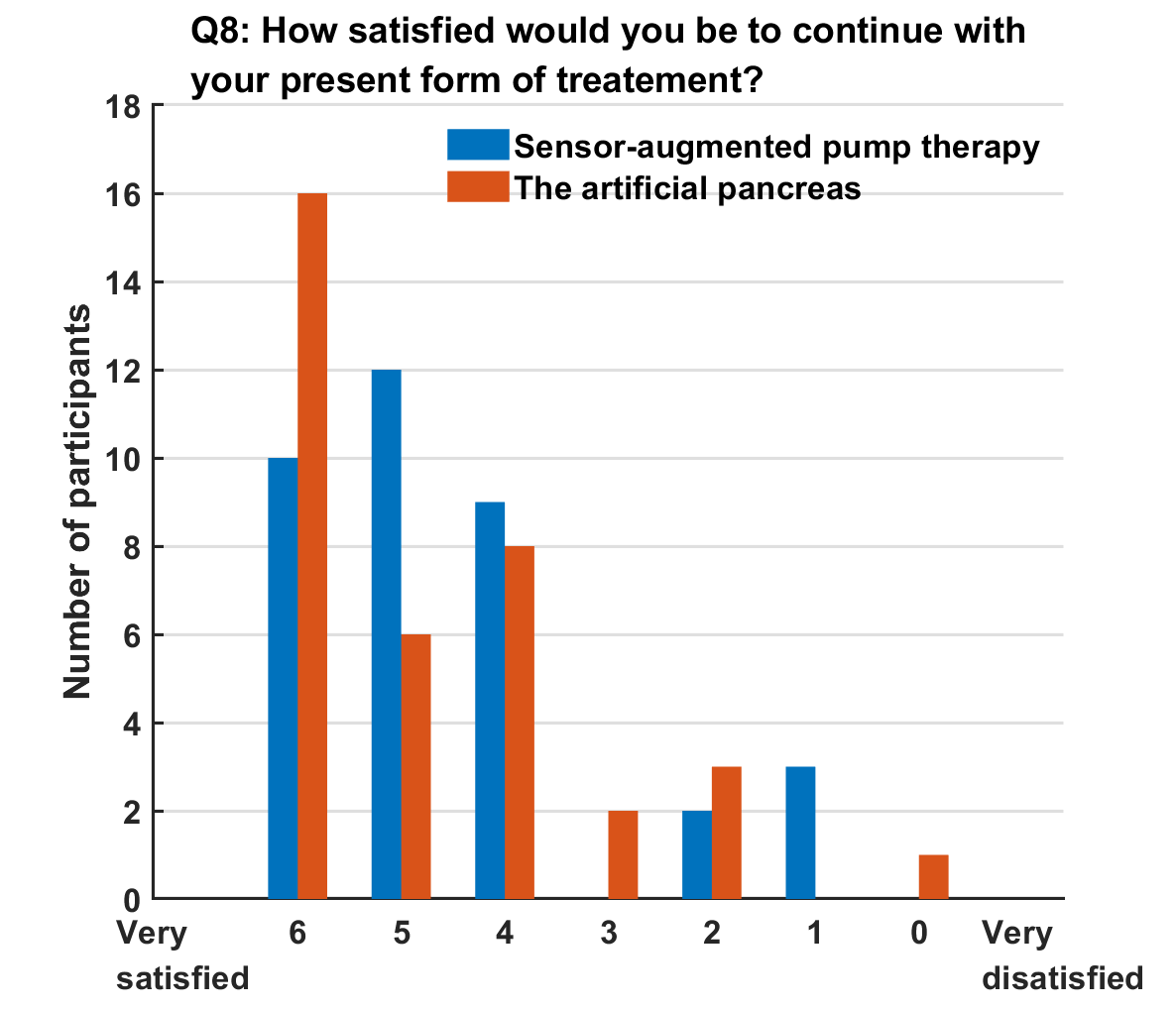

Supplement: Supplemental data [file Supp_Data.zip › Supplementary Appendix.docx]
